# Viral Metagenomics Analysis of Rodents From Two Border Provinces Located in Northeast and Southwest China

**DOI:** 10.3389/fmicb.2021.701089

**Published:** 2022-02-21

**Authors:** Teng Zhao, Yun-qi Dang, Ao-nan Wang, He-ting Gao, Heng-duan Zhang, Dan Xing, Bo-qi Li, Yong-jiu Li, Zhu Liu, Chun-xiao Li

**Affiliations:** ^1^State Key Laboratory of Pathogen and Biosecurity, Institute of Microbiology and Epidemiology, Beijing, China; ^2^College of Life Science and Technology, Mudanjiang Normal University, Heilongjiang, China; ^3^Raohe Customs, Heilongjiang, China

**Keywords:** rodents, viral metagenomics, hantavirus, Beilong virus, phylogenetics

## Abstract

**Objective:**

Wild animal pathogen surveillance will help to understand the next possible pandemic in advance. Rodents, which have close contact with humans, are generally regarded as a key factor for zoonotic disease control. Given the variation in rodent virus composition in diverse ecologies, we conducted a study on the viral infection of rodents of diverse species in different typical environments of Heilongjiang and Yunnan Provinces, located in northeastern and southwestern China, respectively.

**Methods:**

Viral metagenomics sequencing and bioinformatic analysis were performed to determine the different distributions of rodent-borne viruses in typical environments of Heilongjiang and Yunnan Provinces, China. After viral culture and PCR confirmation, genomic and phylogenetic quantitative analysis was performed on the detected hantaviruses (HVs) and Beilong viruses (BeiVs).

**Results:**

Nineteen rodents from three species and 35 rodents from five species of rodents were collected from Heilongjiang and Yunnan Provinces, respectively. Although the number and number of species of rodents trapped in the northeast were fewer than those in the southwest, viruses annotated from rodents in Heilongjiang were more diverse than those in Yunnan. Rodents carried 22 virus families in Heilongjiang and 13 families in Yunnan. Sequences assembled from *Rattus norvegicus* were annotated to the M, L, and S segments of HV, and all were clustered within the Seoul-type hantavirus (SEOV). There were 2 (R81Q, S698T) and 4 (K153R, M168I, I279S, and R1790K) amino acid site substitutions in M and L compared with the versions in the most homologous strains. Two BeiV isolates from *Rattus norvegicus* were closely related to BeiV from brown rats in Hong Kong, with high bootstrap values of >90% in the N segment and > 95% in the L segment. They were further clustered with Tailam virus, forming a distinct group in Paramyxoviridae.

**Conclusion:**

The rodents from Heilongjiang and Yunnan located in northeast and southwest China, respectively, had different viral spectra, and only one-third (10/32) of virus families were detected in both areas. The predominant viruses were HV and BeiV in the *Hantaviridae* and *Paramyxoviridae* families, respectively. Rodent-borne viruses in the same species were similar in different geographic disparate areas owing to their similar close contact with human habitats and human activities. Additional attention should be given to the monitoring of neglected rodent-borne viruses, especially opportunistic viruses with currently low loads.

## Introduction

More than 70% of emerging infectious diseases originate in wild animals, posing serious hazards to public health and societal development around the world (Jones et al., 2008). Rodents are recognized reservoir hosts for many viruses, for example, hantaviruses (HVs). In China, the predominant hosts of HVs are wild *Apodemus agrarius* and domestic *Rattus norvegicus* (Wang et al., 2000; Huchon et al., 2002; Tsoleridis et al., 2016). Rodents have a diverse range of activities, a large number of species, and a high rate of reproduction. Their living space overlaps with human habitats, providing numerous opportunities for contact with humans. In addition, communication between house mice and field mice in suburban farmland can promote the spread of diseases from wildlife to humans. Rodents transmit diseases mainly through direct transmission and indirect transmission. Direct transmission includes rodent bites, exposure to rat secretions, and intake of food contaminated by rodents, and indirect transmission involves vectors such as ticks, fleas, and mites (Liu, 2018).

Metagenomics is a discipline that uses high-throughput sequencing technology to analyze microbial genetic composition from the genome perspective. Viral metagenomics can quickly and accurately identify known virus components and provide hints for unknown virus discovery. Viral metagenomics has been applied to animal, water, and fecal specimens and has facilitated the discovery of several new mammalian viruses, insect viruses, and plant viruses, including a new coronavirus (Appolinaire et al., 2009; Ng et al., 2009; Donaldson et al., 2010; Li et al., 2010). Metagenomics provides a favorable means for the early discovery and exploration of new or unknown pathogens and the pathogen composition in the environment or individuals.

We were interested in the viral spectrum of diverse rodent species in two border provinces with distinct ecologies and environments. Typical areas (Raohe County, Heilongjiang Province; Jinghong city and Ruili city, Yunnan Province) were investigated in this study using the viral metagenomics analysis approach. Heilongjiang Province and Yunnan Province are located in northeast and southwest China, respectively. Both are rich in natural resources but are significantly different in geographical climate and species composition. Heilongjiang Province has a cold temperate and temperate continental monsoon climate, with temperate conifer-broadleaved mixed forest as the main vegetation type. Yunnan Province is dominated by a subtropical plateau monsoon climate, with tropical rainforest and evergreen broad-leafed forest as the main vegetation types (Zhu et al., 2015). Moreover, climate, environment, and population exchange are suitable for rodent-borne disease transmission. This study aimed to determine the difference in rodent abundance and virus spectrum in these extremely diverse environments, which contributed to identifying the pathogen spillover and transmission mechanisms.

## Materials and Methods

### Rodent Trapping

**FIGURE 1:**
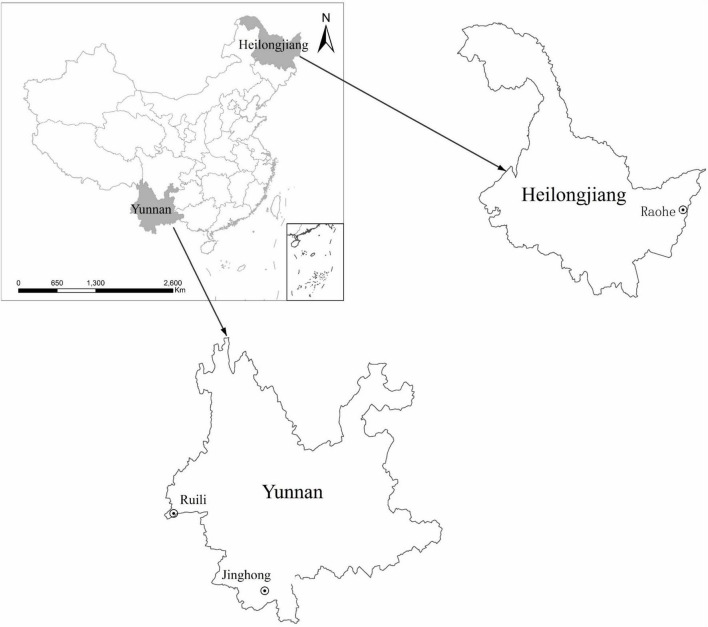
Rodent trapping sites in Northeast and Southwest China.

Rodents were trapped from Raohe County (N 46.79899, E 134.01986) in Heilongjiang Province and from Jinghong city (E 100.79977, N 22.01071) and Ruili city (N 24.01277, E 97.85183) in Yunnan Province from August to September 2019 (Figure 1). The sampling sites were human habitats and nearby farmland. According to morphological characteristic guidance (Zhou and Chu, 2019), skull size, odontoid process, skin color, body length, and tail length, rodent classifications were identified. Organs, including the brain, lung, liver, spleen, kidney, heart, intestine, and stomach, were extracted using sterile procedures, placed into cryotubes with viral preservation solution, and stored at −80°C. All animals were euthanized according to humanitarian principles.

### RNA Extraction

The collected rodent specimens (mixed brain, lung, liver, spleen, kidney, heart, intestine, and stomach specimens) were divided into 11 pools according to sampling sites and species. *Rattus flavipectus* from Yunnan was divided into four subgroups by sex, age, and sampling site (Table 1). Precooled sterile phosphate-buffered saline (PBS) was added at a ratio of 1:10 (w/v). Organs were vortexed, homogenized thoroughly using a homogenizer, and then centrifuged at 10,000 r/min and 4°C for 10 min (rotor: S4-104, Eppendorf 5810R). The supernatant was collected, and the precipitate was added to sterile PBS and vortexed until well mixed. Similar procedures were as follows: centrifugation at 6,000 r/min for 15 min, remixing, 4,000 r/min for 15 min, remixing, and 8,500 r/min for 10 min. The resultant supernatant was centrifuged at 4,5000 r/min in an ultracentrifuge (rotor: RPS65T Hitachi CP100MX) for 1.5 h. After the supernatant was discarded, the pellet was resuspended in sterile PBS, appropriate amounts of DNase I and RNase A were added, the sample was incubated in a water bath at 37°C for 2 h to degrade the cell-free nucleic acids, and then the sample was kept in a water bath at 75°C for 10 min to inactivate DNase I and RNase A.

**TABLE 1:** Groups of collected rodent specimens in Heilongjiang, Northeast China and Yunnan, Southwest China.

Number	Group	Sampling site	Species	Number	Collection date	Remarks
1	RH1	Raohe, Heilongjiang	*Microtus fortis*	9	*22nd August*	
2	RH2	Raohe, Heilongjiang	*Rattus norvegicus*	5	*22nd August*	
3	RH3	Raohe, Heilongjiang	*Apodemus agrarius*	5	*22nd August*	
4	YN2	Ruili, Yunnan	*Rattus flavipectus*	5	*2 on 18th September and 3 on 25th September*	pups
5	YN3	Ruili, Yunnan	*Rattus flavipectus*	8	*4 on 18th September and 4 on 25th September*	female adults
6	YN4	Ruili, Yunnan	*Bandicota indica*	1	*18th September*	
7	YN5	Ruili, Yunnan	*Rattus rattus sladeni Anderson*	5	*25th September*	
8	YN7	Ruili, Yunnan	*Rattus yunnanensis*	1	*25th September*	
9	YN9	Ruili, Yunnan	*Eothenomys miletus*	1	*25th September*	
10	YN10	Ruili, Yunnan	*Rattus flavipectus*	4	*18th September*	male adults
11	YN11	Jinghong, Yunnan	*Rattus flavipectus*	7	*23rd September*	female adults

### Viral Metagenomic Sequencing

Genomic RNAs were quantified in a NanoDrop to detect the integrity of the genomes and ensure that there were minimal impurities and total RNA degradation (Desjardins and Conklin, 2010). The RNA specimens prepared with the RNA enrichment platform were fragmented to 200–300 bp and subjected to reverse transcription to obtain complementary DNA (cDNA). The ends of the obtained cDNA fragments were ligated to Illumina sequencing adaptors for fragment screening and purification using magnetic beads. The obtained target fragment library was enriched and purified with magnetic beads for library construction. The specimens were sent to Beijing Macro and Micro-test Bio-Tech Co., Ltd., for viral metagenomic sequencing using an Illumina NextSeq 500 sequencer.

### Bioinformatic Analysis

The original image data were converted into sequence data through base-calling and stored in a FASTQ format (Cock et al., 2010; Yang et al., 2016). Illumina’s Casava 1.8 software was used for quality control and examination of the GC content distribution. The BWA software was used to remove host data (Li, 2013). The distribution of pathogen species in the sequencing data was determined by screening and species annotation through alignment with the GenBank database of pathogenic nucleic acids. The filtered reads were assembled into contigs and then scaffolds by SPAdes (v3.12.0) (Bankevich et al., 2012; Nurk et al., 2017). The Basic Local Alignment Search Tool (BLAST; version 2.5.0) software was used to align them with sequences in the nucleotide database of the National Center for Biotechnology Information (NCBI) (Altschul et al., 1997). Viral read values under 200 nucleotides were discarded from the subsequent diversity analyses to avoid potential contamination and reduce the possible overestimation of viral diversity. A heatmap was created using the R (3.6.3) package pheatmap, and a principal component analysis (PCA) scatterplot was created using the ggplot2 package. Intersection sizes were illustrated with the UpSetR package. Metagenomic sequencing raw data were deposited in NCBI BioProject under accession number “PRJNA777640.”

### Viral Culture and Reverse Transcription–PCR Sequencing

Potent virus infection specimens were inoculated into 9-day-old specific pathogen-free (SPF) layer embryos for virus isolation. For cultured viruses, reverse transcription–PCR (RT-PCR) was conducted for validation. Total RNA was extracted using a TRIzol reagent (Invitrogen, Carlsbad, CA, United States) and dissolved in 50 μl of RNase-free H_2_O. Beilong virus (BeiV) screening was performed by PCR amplification of a 440-bp fragment of the large (L) gene (primers: LPW9739-F: 5′-GGAGGATTCCCTC ATAGR-GAA-3′ and LPW9741-R: 5′-CTCATATGTATTTACAT TTAAACCA-3′) and a 318-bp fragment of the nucleocapsid (N) gene (primers: LPW10723-F: 5′-TATATGGTTGAGATYCT NATHGA-3′ and LPW10408-R: 5′-CCATKGCRTAGCTCCAD AG-3′). RT-PCR was carried out with one cycle of 50°C for 30 min and 94°C for 3 min, followed by 40 cycles of 94°C for 30 s, 55°C for 30 s, and 72°C for 30 s. HV screening was performed by PCR amplification of a 445-bp fragment of the small (S) gene (primers: S-F: 5′- AAAAGTAGGTGITAYATCYTIACAA TGTGG-3′ and S-R: 5′- GTACAICCTGTRCCIACCCC-3′), a 411-bp fragment of the large (L) gene (primers: L-F: 5′-AATGT GTGGTTCACACATAAGGG-3′ and L-R: 5′-ACCTGTATAAG CACTCTCATCCTG -3′), and a 678-bp fragment of the segment M gene (primers: M-F: 5′-GATACTATGAGGCAGTCCACCC -3′ and M-R: 5′-TGGCTTGACAAACTTTGTAATGTGCC -3′). RT-PCR was carried out with one cycle of 50°C for 30 min and 94°C for 2 min, followed by 35 cycles of 94°C for 30 s, 58°C for 30 s, and 72°C for 30 s.

### Phylogenetic Analysis

Hantavirus and Beilong viruses sequences obtained by PCR were deposited in GenBank (accession no. MW881018-881025) and compared with others via BLAST, and the sequences of the reference strains with the highest homology and the classical virus strains were selected for the alignment of nucleic acid sequences using ClustalW (Aiyar, 2000; Larkin et al., 2007). A phylogenetic tree was constructed by the maximum likelihood (ML) method using the Molecular Evolutionary Genetics Analysis (MEGA) 7.0 software, with 1,000 bootstrap replicates (Kumar et al., 2016).

## Results

A total of 19 rodents in three species (*Microtus fortis, Rattus norvegicus*, and *Apodemus agrarius)* were collected in Raohe, Heilongjiang Province, Northeast China, and 32 rodents were collected in five species (*R. flavipectus, Bandicota indica, Rattus rattus sladeni Anderson, Rattus yunnanensis*, and *Eothenomys miletus*) in Ruili and Jinghong, Yunnan Province, Southwest China.

**FIGURE 2:**
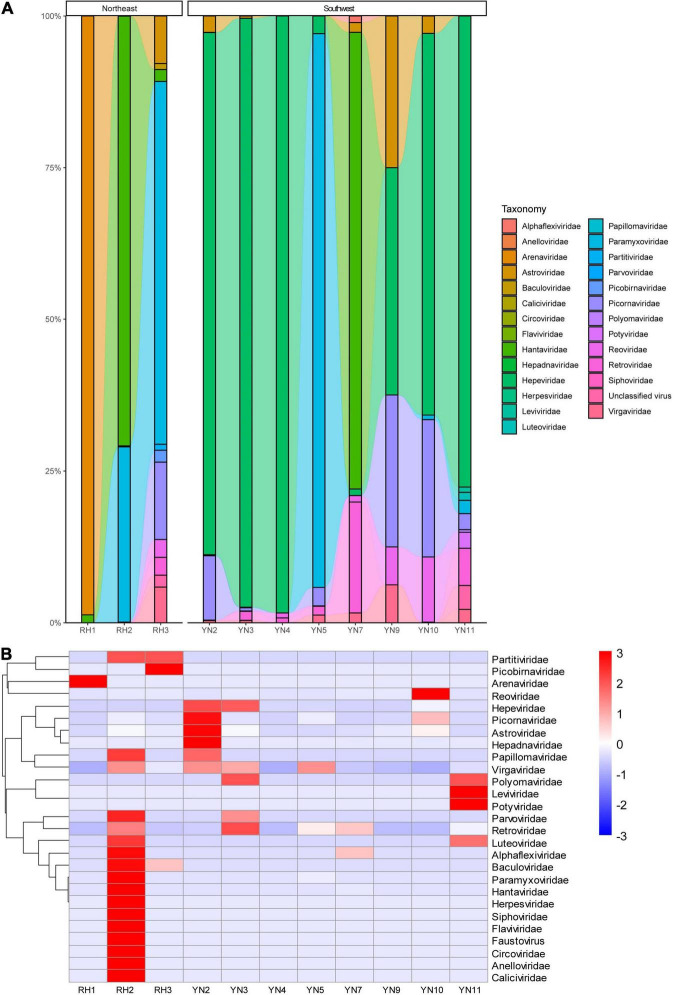
**(A)** Comparative abundance composition of virus reads annotated in rodent specimens in Heilongjiang, Northeast China, and Yunnan, Southwest China. Virus families with low reads are invisible in the bar. **(B)** Heatmap of the viruses annotated in rodents from Northeast and Southwest China.

After removal of the host data, a total of 760,015 reads were obtained from high-throughput sequencing, including 183,659 reads annotated to viruses (13,239 reads in Yunnan and 170,420 reads in Heilongjiang), 568,959 reads annotated to bacteria, and 7,397 reads annotated to fungi. The highest number of virus reads was annotated to *Hantaviridae* and *Paramyxoviridae* from *R. norvegicus* in Heilongjiang, indicating the possible infection of local mice. According to viral hosts, 183,472 (99.8982%) sequences were annotated to vertebrate viruses, 110 sequences (0.0598%) were annotated to plant viruses, and 8 sequences (0.0016%) were annotated to bacteriophages. Annotated virus reads covered 32 families and 62 genera. Sequences from rodents in Heilongjiang were annotated to a total of 22 virus families, with *Hantaviridae, Paramyxoviridae*, and *Arenaviridae* accounting for the top rankings. Sequences in Yunnan were annotated to a total of 13 virus families, ranking with *Hepeviridae, Paramyxoviridae*, and *Picornaviridae* (Figure 2A). Different virus families had preferred rodent hosts. *R. norvegicus* (RH2) had the highest abundance (10 virus families: *Alphaflexiviridae, Anelloviridae, Baculoviridae, Caliciviridae, Circoviridae, Flaviviridae, Hantaviridae, Herpesviridae, Paramyxoviridae*, and *Siphoviridae*). Similarly, *Araneidae* and *Picobirnaviridae* had their highest abundances in *M. fortis* (RH1) *a*nd *A. agrarius* (RH3), respectively. *R. flavipectus* (YN2, YN10, and YN11) was a suitable host for *Astroviridae, Hepadnaviridae, Picornaviridae*, *Reoviridae*, *Leviviridae*, and *Potyviridae* (Figure 2B).

**FIGURE 3:**
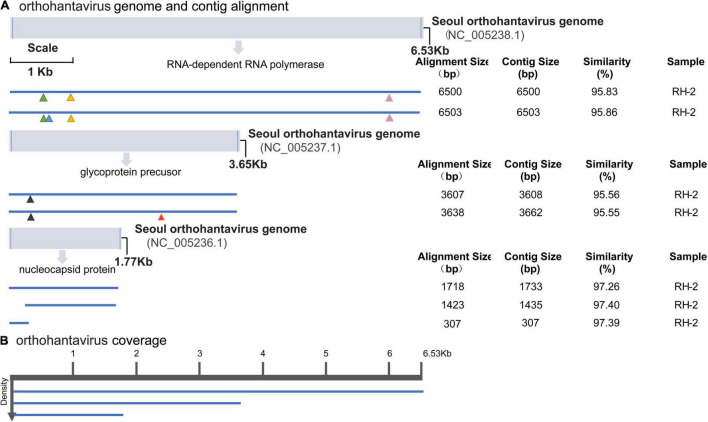
Hantavirus genome organization and mapping of *de novo*–assembled contigs. **(A)** Coordinates are based on the reference sequence number X14736.2. Open-reading frames are represented by cylinders. Genomic RNA is represented by a solid line. Colored triangles mark the amino acid site substitutions. For every sample categorized as infected, the longest contig is shown. Shorter, redundant contigs are not illustrated. Contig size, alignment size, and similarity (%) are indicated. **(B)** Genome coverage after reference-based assembly using Bowtie v2 for one representative sample. Sequence density is indicated on the left.

We focused on the most abundant virus families in all rodent pools, *Hantaviridae* and *Paramyxoviridae* (confirmed as HV and BeiV species). Genome organization and mapping of *de novo*–assembled contigs were performed. HV was detected in the RH2 pool of mixed tissues (brain, lung, liver, spleen, kidney, heart, intestine, and stomach) of 5 *R. norvegicus* animals. The genome of HVs consists of 3 single-stranded, negative-sense RNA segments (L, M, and S) encoding RNA-dependent RNA polymerase, glycoprotein precursor, and nucleocapsid protein, respectively. Single contigs nearly covered the complete genome. Compared with the SEOV standard genome (NC_005236-NC_005238 for the three gene segments), the assembled sequences of HV-RH2-L were approximately 6,500 nt long with 95.83–95.86% nucleotide similarity. There were 4 amino acid site substitutions, K153R, M168I, I279S, and R1790K (marked in colored triangles). The sequences of HV-RH2-M were 3,608–3,662 nt long and consisted of an ORF of 3,402 nt that encoded a putative 1,134 amino acid (aa) glycoprotein precursor with 95.55–95.56% nucleotide similarity. There were 2 amino acid site substitutions, R81Q and S698T (marked in colored triangles). The HV-RH2-S contigs were 307 to 1,733 nt long, encoding the NP protein of 430 aa with 97.26–97.40% nucleotide similarity. The NP protein contained 4 conserved cysteines, and no differences with reference were observed in the amino acid sequence, suggesting low genetic variation in the HV population in Raohe, Heilongjiang (Figure 3).

**FIGURE 4:**
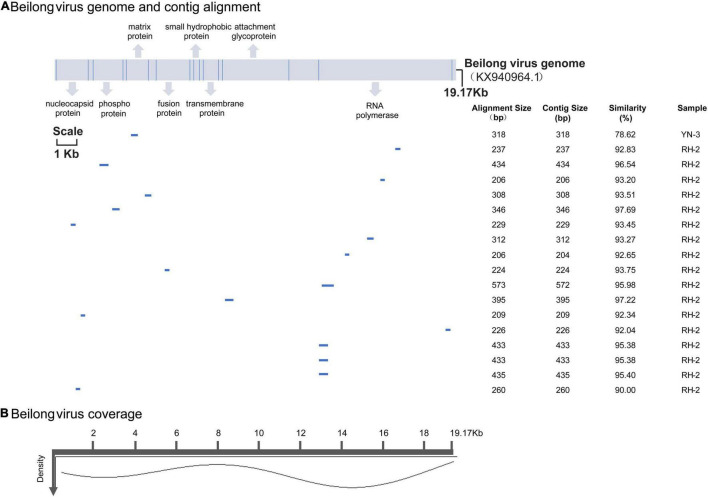
**(A)** BeiV genome and polyprotein organization. Mature proteins are represented by cylinders. Coordinates are based on the reference (KX940964.1). Labels are as described in Figure 3. **(B)** Genome coverage after reference-based assembly using Bowtie v2 for one representative sample.

BeiV belongs to the family *Paramyxoviridae*, genus *Jeilongvirus*. It has an exceptionally large genome (>19 kb) and contains more than eight transcriptional units. BeiV was detected in 2 pools, RH2 and YN3, which contained mixed tissues of 5 *Rattus norvegicus* and 8 *Rattus flavipectus* animals, respectively. Except for a 318-bp contig encoding matrix protein from the YN3 sample with less than 90% nucleotide similarity, the other 17 assembled contigs ranged from 209 to 573 bp with 90.00–97.68% similarity to the reference genome (KX940964.1). With respect to the reference genome, these contigs lacked sense mutations. The coverage indicates the percentage of the genome area covered by an average of reads, while density refers to the number of reads covering the same sequence. Most BeiV contigs covered the area of RNA polymerase (Figure 4).

Ten families, including *Alphaflexiviridae, Astroviridae, Hantaviridae, Herpesviridae, Luteoviridae, Papillomaviridae, Paramyxoviridae, Picornaviridae, Reoviridae*, and *Retroviridae*, were annotated from rodent pools from both Heilongjiang and Yunnan. RH2 and RH3 in Heilongjiang had the same 5 virus families as those in Yunnan. YN2, YN3, YN10, and YN11 were from the same species but different sites, sex, and age, while they had 6 families in common (Supplementary Figure 1). In PCA, due to the high abundance of HV carried by *R. norvegicus* from Raohe County, the HV strains formed an independent branch. Most of the specimens from Yunnan clustered together and were slightly separated from the specimens from Heilongjiang. YN2, YN3, and YN10 were from *R. flavipectus* from Ruili County in Yunnan, and YN11 was from the same rodent species from Jinghong County in Yunnan, which is 700 km distant (Supplementary Figure 2).

**FIGURE 5:**
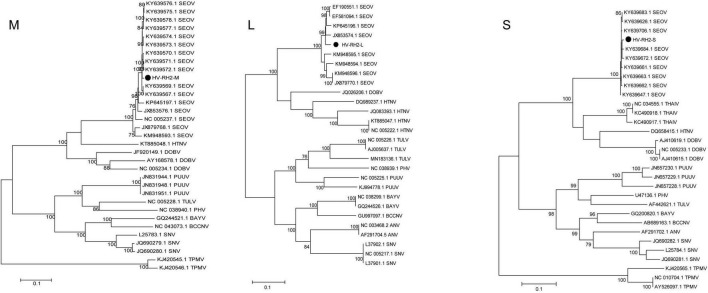
Maximum likelihood phylogenetic tree constructed based on the M, L, and S segments of Hantavirus. Seoul virus (SEOV), Hantaan virus (HTNV), Sin Nombre virus (SNV), Black Creek Canal virus (BBCV), Bayou virus (BAYV), Andes virus (ANDV), Tula virus (TULV), Prospect Hill virus (PHV), Puumala virus (PUUV), New York virus (NYV), Thailand virus (THAIV), Dobrava virus (DOBV), Andes virus (ANV), and Thottapalayam virus (TPMV).

Tissue-grinding fluid from potentially infected RH2 (including RH2-2/2-10/2-12/2-13/2-19) was inoculated into 9-day-old SPF layer embryos for virus isolation. There were four rodents with nine tissue samples (kidney, lung, and spleen) and one rodent with a kidney tissue sample confirmed by RT-PCR as having BeiV and HV infection. To explore the genotypes of the three HV sequences in this study, 14 HV types were selected to construct the ML phylogenetic tree. The ML phylogenetic tree showed that HV-RH2-L, HV-RH2-M, and HV-RH2-S clustered together in SEOV, with > 97% nucleotide similarity with strains isolated from *R. norvegicus* in Shandong, China. *R. norvegicus* was recognized as the major host of SEOV. The similarity between HV-RH2-L and KY639672 was 99.37%, the similarity between HV-RH2-M and JX853574 was 97.97%, and the similarity between HV-RH2-S and KY639575 was 99.21%. There were obvious differences between different HV types. The phylogenetic trees indicated that SEOV strains from Heilongjiang in 2019 clustered together with the strains collected from 2012 to 2015 in Shandong, which is the main epidemic area of HV in China (Figure 5).

**FIGURE 6:**
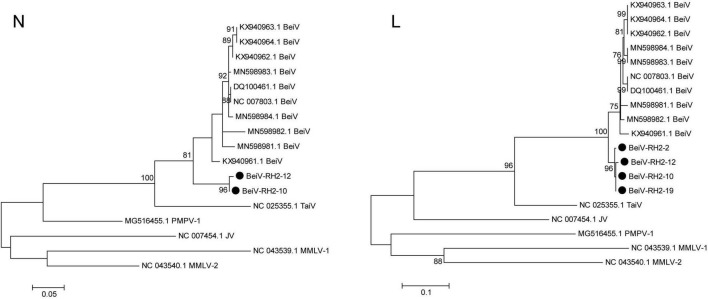
Maximum likelihood phylogenetic tree constructed based on the N and L segments of Beilong virus. Beilong Jeilongvirus (Beilong virus, BeiV), Tailam Jeilongvirus (Tailam virus, TaiV), Lophuromys Jeilongvirus 1 (Mount Mabu Lophuromys virus 1, MMLV-1), Lophuromys Jeilongvirus 2 (Mount Mabu Lophuromys virus 2, MMLV-2), Mydes Jeilongvirus (Pohorje Myodes paramyxovirus 1, PMPV-1), and Jun Jeilongvirus (J-virus, JV).

A total of 4 BeiV sequences covering the N and L segments were acquired after PCR sequencing. BeiV belongs to the genus Jun *Jeilongvirus* (J-virus, JV). Phylogenetic analyses showed that two N gene (BeiV-RH2-10 and BeiV-RH2-12) sequences were clustered together, with 90.60 and 91.21% similarity to KX940961. Four L genes of BeiV sequences in Raohe, Northeast China, formed a distinct subgroup with MN598982 isolated from *R. norvegicus* in Hankou, Central China. They were clustered with high bootstrap support of > 95%. BeiV sequences from *R. norvegicus* in Northeast China were clustered together in a branch with those from *R. norvegicus* in Central and Southern China, suggesting that brown rats are natural reservoirs of BeiV. In addition, these viruses were further clustered with Tailam virus (TaiV), with high bootstrap support of >90%, forming a distinct group in *Paramyxoviridae* (Figure 6).

## Discussion

Yunnan in Southwest China and Heilongjiang in Northeast China, which border other countries, are ecologically diverse and rich in animal resources. Yunnan has a subtropical plateau monsoon climate with tropical rainforest and evergreen broad-leafed forest, while Heilongjiang has a cold temperate and temperate continental monsoon climate with temperate conifer-broadleaved mixed forest. Rodents are common hosts for a variety of viruses, and their habitats have a high overlap with human habitats. The dominant rodent species in these two provinces are *R. norvegicus* and *R. flavipectus*. The highest number of viral genera and families were annotated from *R. norvegicus* in Heilongjiang Province. *R. norvegicus* is a dominant species and an important viral host in Raohe County (Wang et al., 2020). Rodents bring great challenges for the prevention and control of infectious diseases due to their activities (Mills, 2006; Bordes et al., 2015).

The rodents from Heilongjiang and Yunnan Provinces had different viral spectra, and only one-third (10/32) of virus families were detected. Sequences from rodents in Heilongjiang were annotated to a total of 22 virus families, with *Hantaviridae, Paramyxoviridae*, and *Arenaviridae* accounting for the top rankings. Sequences from Yunnan rodents were annotated to a total of 13 virus families, with *Hepeviridae, Paramyxoviridae*, and *Picornaviridae* accounting for the top rankings. Rodents from two border provinces located in Northeast and Southwest China had different viral spectra, and the virus spectra of the same *R. flavipectus* species were quite different between Ruili and Jinghong County, which are separated by a long distance, to some extent, which could be explained by rodent sex, age, and activity area. The overlapping virus spectrum between two border provinces located in Southwest China and Northeast China indicated that both the ecological environment and the living habitat contributed to the virus composition, which reflected the strong influence of human activities.

Even though the number and species of rodents trapped in Heilongjiang Province were less than those in Yunnan Province (19 vs. 32 in number, 3 vs. 5 in species), viruses annotated from rodents in Heilongjiang were more diverse than those in Yunnan (22 vs. 13 virus families). Most virus families detected in this study were from *R. norvegicus* and *A. agrarius*, which were the predominant hosts of Hantaan virus (HTN)-type and Seoul-type HVs (SEOVs) (Zeier et al., 2005). Both rodent species were abundant and widely distributed in North China. They had a wider range of predation and activity than the predominant species *R. flavipectus* in the southwest. Additionally, the limited number and the species of rodents analyzed could bias the results, which would be improved with a large-scale monitoring plan.

*Hantaviridae* strains were annotated in all three rodent species from Heilongjiang, and their abundance was higher than that in Yunnan. In addition, *Hantaviridae* was found in higher abundance in *R. norvegicus* than in *M. fortis* and *A. agrarius.* Phylogenetic trees constructed based on the M, L, and S segments indicated that the HV genotype carried by *R. norvegicus* was SEOV, which is consistent with the finding of previous studies showing that local *R. norvegicus* is a major host of the SEOV genotype (Xiao et al., 2013; Han et al., 2015). Envelope glycoproteins, including their neutralizing antigen sites, receptor-binding sites, fusion peptides, and hemagglutination sites, play an important role in the pathogenicity of the virus. A glycoprotein precursor is generated by the M fragment and then cleaved into two proteins, G1/G2, by the signal peptidase in the cell during translation. Even though there were 2 (R81Q and S698T) amino acid site substitutions found in the M segment, the pathogenicity was genetically stable. The phylogenetic trees indicated that SEOV strains from Heilongjiang in 2020 clustered together with the strains collected from 2012 to 2015 in Shandong, which is the main epidemic area of HV in China, suggesting that the SEOV genotype was dominant in the north and had an expanding trend.

The abundance of BeiV in the *Paramyxoviridae* family annotated in all specimens was second only to that of HV. This was the first study to detect BeiV in rodents in Heilongjiang Province (Chen et al., 2020). The similarity of the BeiV sequence in this study to the known BeiV sequences was lower than 95%, suggesting that it might be a new genotype. In addition, *R. norvegicus* might be a potential host of BeiV, which was widely distributed throughout Heilongjiang Province. In Yunnan, Southwest China, TaiV was detected in *R. flavipectus* and has a close genetic relationship with BeiV and belongs to *Paramyxoviridae* (Woo et al., 2011; Vanmechelen et al., 2018). TaiV was first found in Sikkim rats and currently cannot infect humans. However, there is a possibility that continuous evolution will yield pathogenic mutant strains.

*Hepeviridae* was annotated in all eight pools from Yunnan. *Hepeviridae* was most abundant in *R. flavipectus*, perhaps because *R. flavipectus* was common in Yunnan and more likely to have close contact with humans than other rodents. A study showed that the hepatitis E virus IgG-positive rate in *R. flavipectus* was 19.9% (34/171) in South China in 2012 (Wei et al., 2013). These results suggest that if preventive measures are not effectively implemented, transmission of hepatitis E between rodents and humans might occur.

A small number of plant viruses and insect viruses, including *Discistroviridae*, *Baculoviridae, Virgaviridae, Betaflexiviridae, Partitiviridae, Luteoviridae*, and *Potyviridae*, were also annotated probably because some rodent species feed on plants and insects or due to contamination in the natural environment during their activities. Even though viral diversity was adjusted with analytical methods, there was still inevitable bias due to the limitation of the rodent sampling size. A large-scale monitoring plan has been implemented to compensate for these shortcomings.

## Conclusion

The rodents from two border provinces located in Northeast and Southwest China had different viral spectra. HV and BeiV were the predominant viruses in Heilongjiang, Northeast China, while hepatitis E virus was widely distributed in Yunnan, Southwest China. *R. norvegicus*, which was the preferred rodent host for 10 detected virus families, such as HV, had an abundant and wide distribution in Heilongjiang Province. It carried more species of viruses than the predominant species *R. flavipectus* in Yunnan Province. The overlapping virus spectrum between two border provinces located in Northeast and Southwest China indicated that both the ecological environment and the living habitat environment contributed to the virus composition, which reflected the strong influence of human activities.

## Data Availability Statement

Metagenomic sequencing raw data were deposited in the NCBI BioProject (https://www.ncbi.nlm.nih.gov/bioproject) with accession number “PRJNA777640.” Hantavirus and Beilong virus sequences were deposited in GenBank (https://www.ncbi.nlm.nih.gov/genbank) with accession numbers “MW881018–881025”.

## Ethics Statement

The animal study was reviewed and approved by Ethics Committee of Beijing Institute of Microbiology and Epidemiology.

## Author Contributions

TZ, Y-QD, and A-NW carried out the rodents sampling and molecular genetic studies and participated in the virus isolation. TZ drafted and revised the manuscript. TZ and H-TG analyzed the data and built the figures in both original and revised versions. H-DZ, DX, and B-QL performed the experiments. Y-JL, ZL, and C-XL conceived and designed the study. All authors read and approved the final manuscript.

## Conflict of Interest

The authors declare that the research was conducted in the absence of any commercial or financial relationships that could be construed as a potential conflict of interest.

## Publisher’s Note

All claims expressed in this article are solely those of the authors and do not necessarily represent those of their affiliated organizations, or those of the publisher, the editors and the reviewers. Any product that may be evaluated in this article, or claim that may be made by its manufacturer, is not guaranteed or endorsed by the publisher.

## Supplementary Material

Supplementary Figure 1 Intersection size of virus reads annotated in rodent specimens in Heilongjiang, Northeast China, and Yunnan, Southwest China. The line of different points indicates the existence of intersections, and number of specific intersections can be seen in the bar chart above. A single black dot indicates that the sample had data. The intersection size bar above is the unique numeric size of the virus family, and the parallel set size bar is the total numeric size of the virus family. Sequences from *M. fortis* (RH1), *R. norvegicus* (RH2), and *A. agrarius* (RH3) were annotated to 2, 20, and 10 virus families, respectively, in which 1, 13, and 6 families, respectively, were unique in their own group. In Yunnan, sequences from *R. flavipectus* (YN2), *R. flavipectus* (YN3), *B. indica* (YN4), *R. rattus sladeni Anderson* (YN5), *R. yunnanensis* (YN7), *E. miletus* (YN9), *R. flavipectus* (YN10), and *R. flavipectus* (YN11) were annotated to 7, 8, 3, 6, 7, 5, 6, and 9 families, respectively, in which 1, 1, 0, 1, 0, 0, 0, and 2 families, respectively, were unique in their own group.

Supplementary Figure 2 Principal component analysis of rodents in Heilongjiang, Northeast China, and Yunnan, Southwest China. *R. flavipectus* (YN2), *R. flavipectus* (YN3), and *R. rattus sladeni Anderson (YN5)* in Yunnan were close to each other. *B. indica (YN4)*, *R. yunnanensis (YN7)*, *E. miletus (YN9)*, and *R. flavipectus* (YN10) of Yunnan and *M. fortis* (RH1) and *A. agrarius (RH3)* of Raohe County clustered together.
