# Supplementary figures and images for: Viral Metagenomics Analysis of Rodents From Two Border Provinces Located in Northeast and Southwest China
Source: Front Microbiol. 2022 Feb 21;12:701089. doi: 10.3389/fmicb.2021.701089 (PMC8899188; doi:10.3389/fmicb.2021.701089)

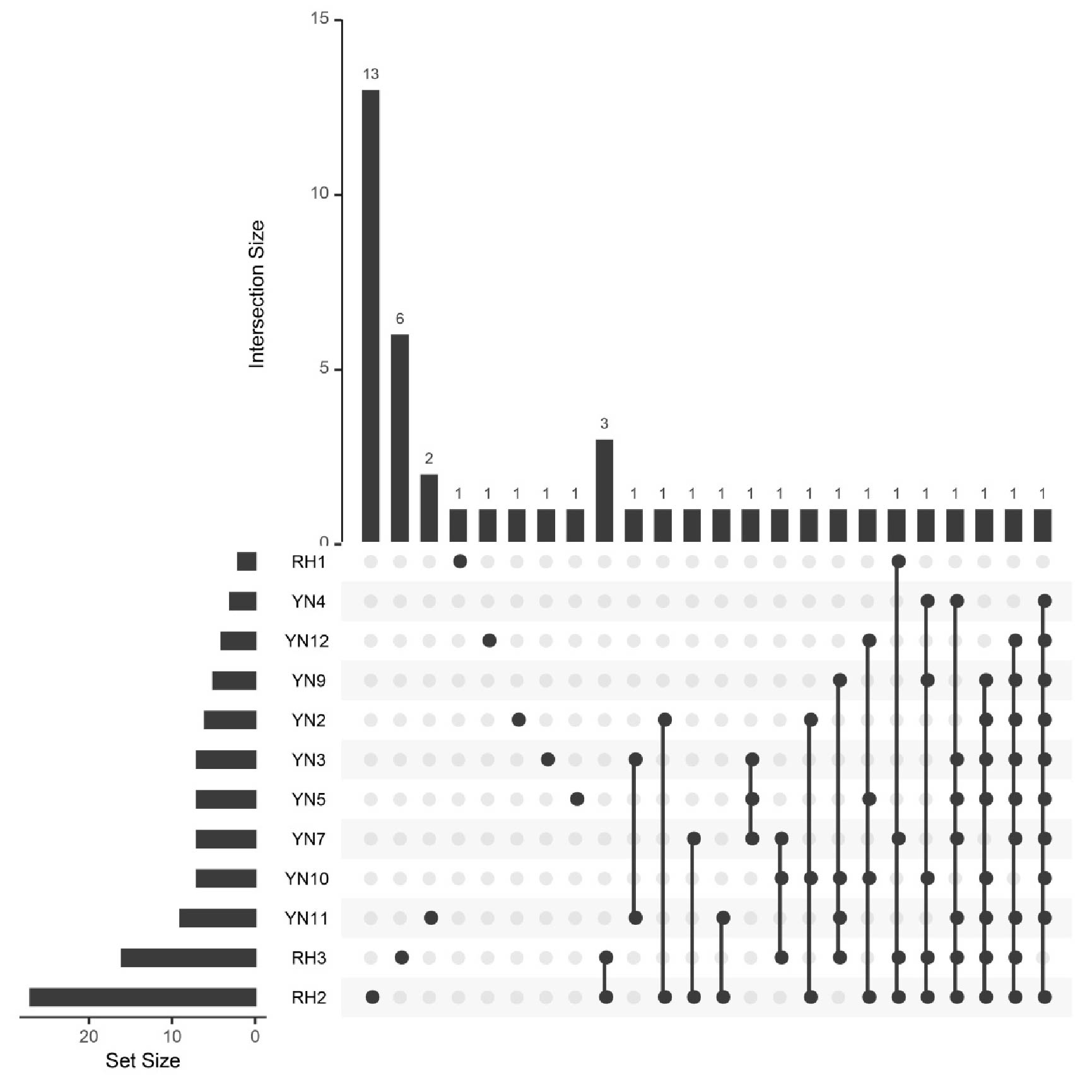

Supplement: Supplementary Figure 1 — Intersection size of virus reads annotated in rodent specimens in Heilongjiang, Northeast China, and Yunnan, Southwest China. The line of different points indicates the existence of intersections, and number of specific intersections can be seen in the bar chart above. A single black dot indicates that the sample had data. The intersection size bar above is the unique numeric size of the virus family, and the parallel set size bar is the total numeric size of the virus family. Sequences from M. fortis (RH1), R. norvegicus (RH2), and A. agrarius (RH3) were annotated to 2, 20, and 10 virus families, respectively, in which 1, 13, and 6 families, respectively, were unique in their own group. In Yunnan, sequences from R. flavipectus (YN2), R. flavipectus (YN3), B. indica (YN4), R. rattus sladeni Anderson (YN5), R. yunnanensis (YN7), E. miletus (YN9), R. flavipectus (YN10), and R. flavipectus (YN11) were annotated to 7, 8, 3, 6, 7, 5, 6, and 9 families, respectively, in which 1, 1, 0, 1, 0, 0, 0, and 2 families, respectively, were unique in their own group. [file Image_1.JPEG]

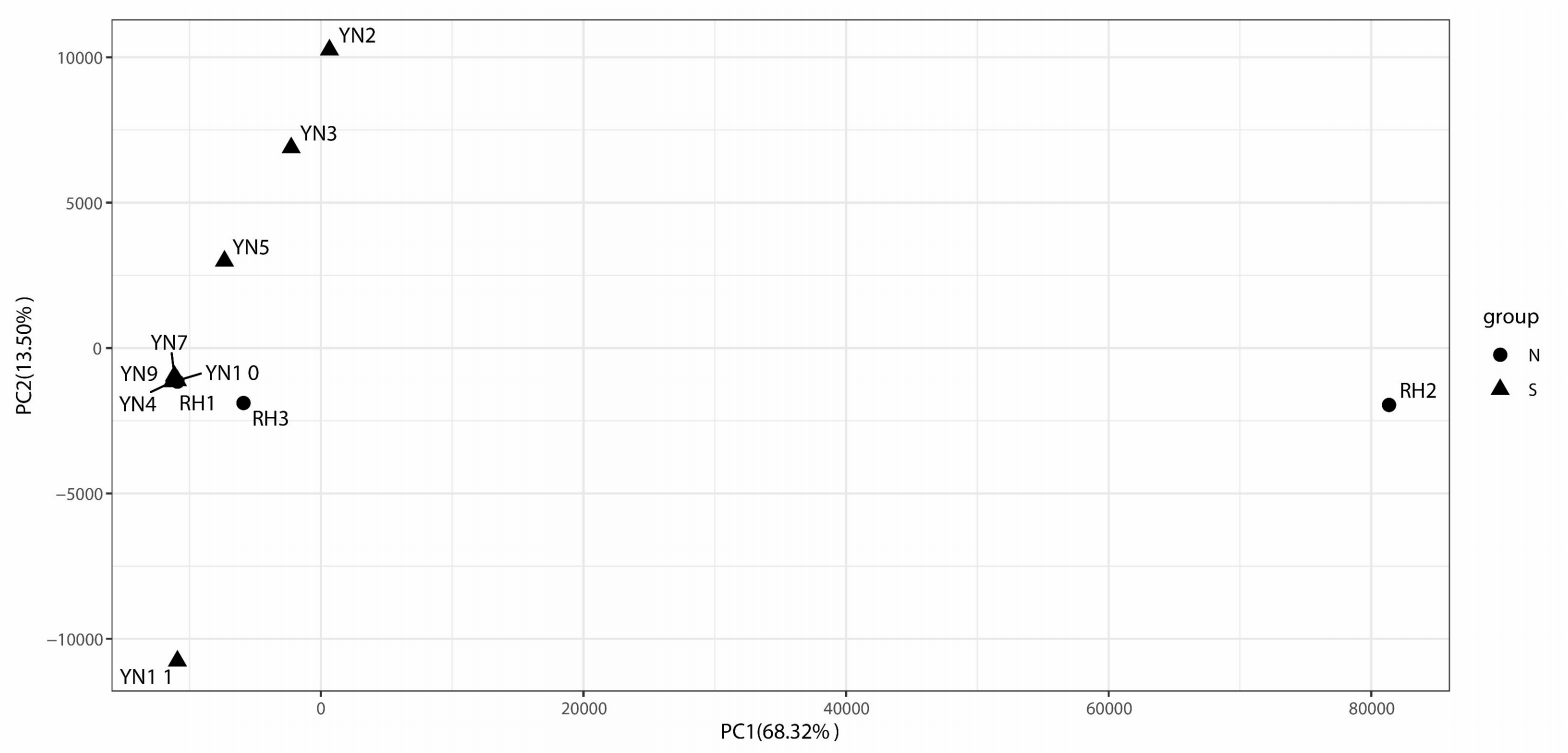

Supplement: Supplementary Figure 2 — Principal component analysis of rodents in Heilongjiang, Northeast China, and Yunnan, Southwest China. R. flavipectus (YN2), R. flavipectus (YN3), and R. rattus sladeni Anderson (YN5) in Yunnan were close to each other. B. indica (YN4), R. yunnanensis (YN7), E. miletus (YN9), and R. flavipectus (YN10) of Yunnan and M. fortis (RH1) and A. agrarius (RH3) of Raohe County clustered together. [file Image_2.JPEG]
